# Association Between PM_10_ and O_3_ Levels and Hospital Visits for Cardiovascular Diseases in Bangkok, Thailand

**DOI:** 10.2188/jea.JE20080047

**Published:** 2009-07-05

**Authors:** Dongruethai Buadong, Wanida Jinsart, Ikuko Funatagawa, Kanae Karita, Eiji Yano

**Affiliations:** 1 Interdepartment Graduate Program in Environmental Science, Graduate School, Chulalongkorn University, Bangkok, Thailand; 2 National Center of Excellence for Environmental and Hazardous Waste Management, Chulalongkorn University, Bangkok, Thailand; 3 Department of General Science, Faculty of Science, Chulalongkorn University, Bangkok, Thailand; 4 Department of Hygiene and Public Health, Teikyo University School of Medicine, Tokyo, Japan; 5 Department of Hygiene and Public Health, Kyorin University, School of Medicine, Tokyo, Japan

**Keywords:** PM_10_, ozone, cardiovascular diseases, hospital visits, Bangkok, air pollution

## Abstract

**Background:**

The association between air pollution and cardiovascular diseases is well known, but previous studies only assessed mortality and hospital admissions in North America, Europe, and Northeast Asia. Few studies have been conducted in less-developed countries in regions with a tropical climate. This study evaluated whether short-term exposures to fine particulate matter (PM_10_) and ozone (O_3_) were associated with hospital visits for cardiovascular diseases (CVD; ICD-10th, I00–I99) in central Bangkok, Thailand.

**Methods:**

Data from hospital records were obtained from 3 major government hospitals. All hospital visits were stratified by age group and category of CVD. Daily PM_10_ and O_3_ levels reported by the Pollution Control Department from April 2002 to December 2006 (1736 days) were used in a time-series analysis with a generalized additive model procedure.

**Results:**

Exposure on the previous day to PM_10_ and O_3_ had a positive association with hospital visits for CVD among elderly (≥65 years) individuals. The increase in CVD hospital visits in this age group was 0.10% (95% CI, 0.03–0.19) with a 10 µg/m^3^ increase in PM_10_, and 0.50% (95% CI, 0.19–0.81) with an increase in O_3_.

**Conclusions:**

In central Bangkok, a short-term association was observed between increases in daily levels of PM_10_ and O_3_ and the number of daily emergency hospital visits for CVD, particularly among individuals aged ≥65 years.

## INTRODUCTION

In recent decades, epidemiologic studies conducted worldwide have shown that short- and long-term exposure to air pollutants, especially particulate matter, is associated with a consistently higher risk for respiratory and cardiovascular events, including heart attacks and stroke deaths.^1^^–^^7^ The Bangkok metropolitan area has a very high population density (4051 persons per km^2^) and 6.12 million registered motor vehicles. Problems related to traffic-related air pollution in Bangkok are becoming more frequent because of the limited number of transport routes and the rapidly increasing number of vehicles on the roads. High concentrations of particulate matter with a diameter of less than 10 µm (PM_10_) from automobile exhaust and a secondary pollutant, ozone (O_3_), may cause health problems. Bangkok air quality has been monitored by 32 Pollution Control Department (PCD) monitoring stations for several years. Between 2004 and 2006 the daily average PM_10_ and O_3_ concentrations in some areas were higher than the National Ambient Air Quality Standard. There have been many reports implicating PM_10_ and O_3_ as risk factors for heart disease.^4^^–^^7^ Kodavanti et al^8^ found an association between combustion particles and both reduced heart rate variability (HRV) and increased fibrinogen levels in rats. These relations have been confirmed in several human studies, which have shown that airborne particles are associated with increased plasma viscosity,^9^^–^^11^ decreased HRV,^12^^–^^14^ and the onset of myocardial infarction.^7^^,^^9^ Ozone, an oxidant gas, can cause respiratory tract damage that may induce pulmonary inflammation and edema^10^^,^^15^; it has also been found to have a direct bradycardiac effect in animal studies.^16^ Similar to PM_10_, exposure to O_3_ in humans has been associated with a decrease in HRV^13^ and an increase in the risk of hospitalization for heart disease.^17^

Cardiovascular disease (CVD) is the most common cause of morbidity and mortality in the developed world. Accumulating evidence shows that traffic-induced air pollution increases damage to the heart. However, the results of experimental studies of the mechanisms involved are inconclusive.^18^^–^^21^ There have been several reports worldwide on the short-term effects of air pollution and the increased risk of hospital admission or death from CVD^22^^–^^26^; however, almost all these epidemiologic studies have been performed in Europe^24^^,^^26^ and North America.^1^^,^^2^

In Thailand, PM_10_ was found to be associated with increased mortality in Bangkok.^25^ An association was also reported between air pollution and both chronic respiratory symptoms and impaired respiratory function among traffic policemen^27^^,^^28^ and their families.^29^^,^^30^ Thus, although previous studies have found a statistically significant relation between the chronic respiratory effects of ambient PM_10_ in Bangkok, little has been reported on the association with hospital visits for CVD after exposure to fluctuating PM_10_ and O_3_ concentrations. Such a study would provide further insight into the health effects of air pollution in a tropical developing country, where seasonal patterns of illness differ from those in Western countries. In the present study, we examined the associations between daily PM_10_ and O_3_ concentrations and daily hospital visits for CVD.

## METHODS

### Air quality and meteorological data

**Figure 1.**
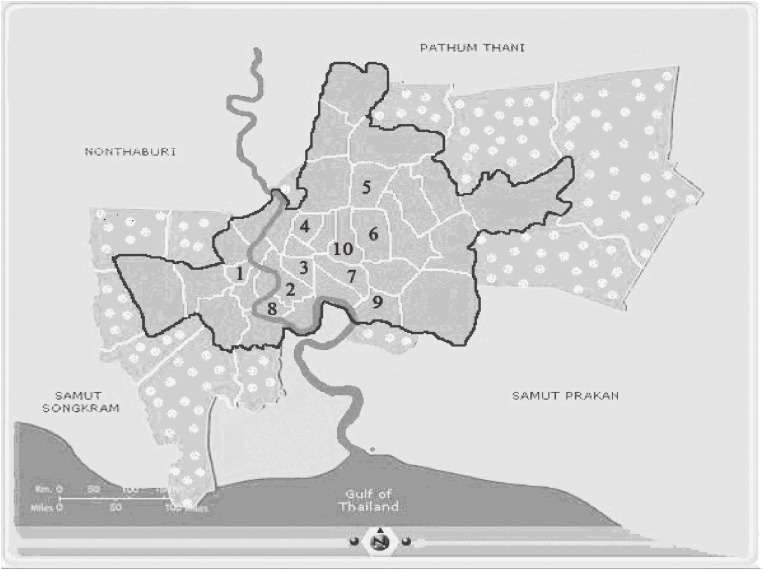
Map of Bangkok: 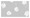 Unstudied area; 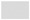 Studied area. Nos. 1–10 indicate the locations of the Pollution Control Department (PCD) ambient monitoring stations: (1) Thonburi Power Substation, Intrapitak Road; (2) 22 Odien Circle, Sampantawong; (3) Ministry of Science and Technology; (4) Dindaeng-National Housing Authority; (5) Chokchai 4 Police Box; (6) Land and Transport Department; (7) Chulalongkorn Hospital. (8) Rat-Burana Post Office; (9) Thai Meteorological Department, Bangna; and (10) Chandrakasem Rajabhat University; Jatujak (Stations 1–7 for PM_10_ and 1–10 for Ozone)

Air quality data in Bangkok were obtained from network monitoring stations of the Pollution Control Department.^31^ From 2002 to 2006, daily PM_10_ concentrations at site 4 (Figure 1) were measured by using a tapered element oscillating microbalance, and data from other sites were measured by using a beta-attenuation mass monitor. (www.aqnis.pcd.go.th/station/allstation.htm)

Ozone was measured by ultraviolet absorption photometry. We used the daily (24-hr) data for PM_10_ at 7 of 13 sites and the average daily (1-hr) O_3_ at 10 of 11 fixed ambient monitoring Pollution Control Department stations in central Bangkok (Figure 1); only stations with data for more than 75% of the entire study period were included in the analysis. Daily weather data from April 2002 and December 2006 were obtained from the Bangkok Meteorology Department. These data included daily average temperature, dew point, wind speed, and daily rainfall.

### Hospital data

Data on hospital visits between 2000 and 2006 were collected using the records of first acute cardiovascular diseases from the 3 government hospitals in Bangkok ie, Ramathibodi, Siriraj, and Chulalongkorn hospitals to represent the population of central Bangkok. The Primary Care Unit System, which requires a fee of only 30 Thai baht (US$ 0.80) per visit for low-income patients, was launched in 2001.^32^ During the period of the introduction of this fee, in the beginning of 2002, there was an unusual fluctuation in the numbers of hospital visits; therefore, we excluded data from before April 2002. Daily counts of total hospital visits were aggregated by age, sex, patient address (zip code), date of hospital visit, and first diagnosis of CVD (ICD-10th code I00–I99).

### Study subjects

Bangkok is divided into 50 districts, with a total registered population in 2007 of 6.6 million.^33^ In these 50 districts, the residential zip codes range from 10002 to 10800. The study subjects were selected from only the 25 innermost districts of Bangkok, where 2 693 292 residents live (Figure 1). The total number of hospital visits for CVD between April 2002 and December 2006 was 33 458. The study protocol was approved by the Institutional Review Board of the Faculty of Medicine; Chulalongkorn University reviewed the protocol based on the international guidelines for human research protection and ICH/GCP.

### Statistical analysis

Time-series data on health outcome and air pollutants were analyzed for the period from 1 April 2002 to 31 December 2006. The Poisson regression model was applied, and the relative risk of a hospital visit was estimated with regression, after controlling for seasonal pattern, effect of the day of week, temperature, and dew point. This was done to control for factors beside air pollutants, which vary on a daily basis and might explain variations in daily hospital visits. We used the Loess smoothing technique to adjust for temperature and dew point. This is a flexible nonparametric modeling tool that can accommodate nonlinear and nonmonotonic patterns between time and health outcome. We also accounted for possible serial correlation in daily hospital visits by using a locally weighted smoothing function of the daily visit count over time.^34^ The smoothing parameters were selected by optimizing the generalized cross-validation criterion. Generalized additive models (GAM) were applied to identify predictor–response relationships among the many types of data, without using a specific model. GAM combine the ability to explore many nonparametric relationships simultaneously with the distributional flexibility of generalized linear models.^35^ Time-series analysis reduces the potential impact of confounding factors and other risk factors that do not vary significantly day-to-day. This model is suitable for exploring the short-term health effects of daily average levels of air pollutants.^36^^,^^37^ The dependent variable was the natural logarithm of the expected hospital visit count, and the regression coefficients were the natural logarithms of the rate ratio. The GAM was estimated as follows.

$$\begin{array}{l} \log\{E(Y)\}=\beta \mathrm{intercept}+\beta \mathrm{PM}+\beta \mathrm{DOW}\\ +\beta \mathrm{Holiday}+\beta \mathrm{Season}\\ +f\mathrm{Temperature}+f\mathrm{Dew point}+f\mathrm{Day} \end{array}$$

Where,

Y=daily number of hospital visitsβ=regressioncoe fficientsPM=primary parameter pollutants(PM10 or O3)DOW=day of week(seven categories:Monday to Sunday)E=expectationf=Loess smoothing function

The associations between daily levels of pollutants and hospital visit variables (Tables 1, 2) were analyzed. Individual lag pollutant exposures on the concurrent day (lag 0), previous day (lag 1), and the 2-day and 3-day averages (lag 0+1 and lag 0+1+2, respectively) were examined. PM_10_ and O_3_ were fitted as linear terms. This statistical analysis provided a relative risk estimate for PM_10_ and O_3_ with a 95% confidence interval (CI).^38^^,^^39^ The relative risks of CVD subsets associated with a 10-µg/m^3^ increase in PM_10_ and O_3_ are presented as the percentage change in daily hospital visits.

**Table 1.** Daily average PM_10_ and O_3_ and related meteorological data for Bangkok from April 2002 to December 2006

Daily average variables	Mean	Minimum	Maximum
24-hour PM_10_ (µg/m^3^)	48.9	19.3	154.9
Hourly O_3_ (ppb)	14.4	3.2	41.9
Dew point (°C)	23.7	11.4	28.1
Wind (km/h)	4.1	0.0	12.1
Temperature (°C)	29.1	21.6	39.9

*National Ambient Air Quality Standards (NAAQS) of 0.10 ppm averaged over 1 hour and 0.07 ppm averaged over 8 hours.

**Table 2.** Descriptive data on hospital visits for cardiovascular diseases (I00–I99)

Variables	Number of visits (cases)	Mean number of visits (cases/day)	Range of cases/day (min–max)
1) All, I code (I00–I99)	33 458	19	0–50
​ 1.1) Age < 15 years	681	1	0–4
​ 1.2) Age 15–64 years	16 710	10	0–25
​ 1.3) Age ≥ 65 years	16 067	10	0–23
2) Arrhythmia (I46–I49)	1876	1	0–8
3) MI (I21)	2566	2	0–8
4) IHD (I20–I25)	10 158	6	0–19

MI, Myocardial Infarction; IHD, Ischemic Heart Disease

## RESULTS

### Air quality and meteorological data

The data for daily average PM_10_ and O_3_ concentrations for more than 75% of the entire study period were obtained from an average of 7 of 13 stations for PM_10_ and 10 of 11 stations for O_3_. The mean level (range) of daily PM_10_ and daily O_3_ (1-hr) was 48.9 µg/m^3^ (19.3–154.9 µg/m^3^) and 14.4 ppb (3.2–41.9 ppb), respectively. The daily weather data showed temperature seasonality: daily average temperatures were lower in November, December, and January. The mean (range) of daily temperature, dew point, and wind speed during the study period were 29.1 °C (21.6–39.9 °C), 23.7 °C (11.4–28.1 °C), and 4 km/h (0–12 km/h), respectively (Table 1). Normally, high summer temperatures occur during the period from February through May, and the rainy season is between May and October. These patterns and seasonal definitions are consistent with those specified by the Meteorology Department of Thailand.^40^

**Figure 2.**
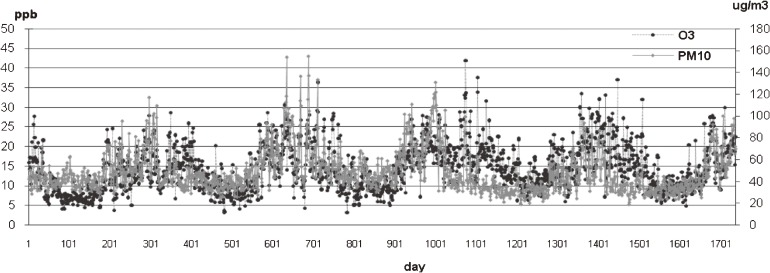
Daily averages of ambient air pollutants (O_3_ and PM_10_ levels) in central Bangkok, from 1 April 2002 to 31 December 2006. (Total number of days = 1736)

Ozone is a photochemical reaction product formed in heavy traffic areas. Production of this co-pollutant is related to many primary pollutants such as hydrocarbons, CO, and NO_x_. An urban environment with high levels of air pollutants can result in high O_3_ formation.^41^ Ambient daily O_3_ and PM_10_ profiles from 2002 to 2006 are shown in Figure 2. The colinearity between PM_10_ and O_3_ were significant in pairwise correlation tests (*r*^2^ = 0.25, *P* < 0.001). The ambient air quality data for 2005 showed that although PM_10_ decreased, O_3_ continued to increase, suggesting that sources other than PM_10_ influence O_3_ formation. However, there was a positive association between daily number of hospital visits and air pollution levels.

### The association between PM_10_ and hospital visits for cardiovascular diseases

The descriptive data on hospital visits for CVD are summarized in Table 2. No significant association was found between PM_10_ exposure and total visits for CVD (or for arrhythmia, MI, or IHD), either on the concurrent day (lag 0) or the previous day (lag 1). However, after controlling for covariate factors, daily PM_10_ concentration was positively associated with hospital visits for CVD among elderly (≥65 years) individuals. Among this group, a 0.10% (95% CI, 0.03–0.19) increase in visits for CVD was associated with a 10-µg/m^3^ increase in PM_10_. The 2-day average PM_10_ concentration was associated with a 0.09% (95% CI, 0.00–0.20) increase in hospital visits for CVD among elderly patients for each 10-µg/m^3^ increase in PM_10_ (Table 3).

**Table 3.** Percentage change in daily hospital visits for cardiovascular diseases (CVD) by 10-µg/m^3^ increase in PM_10_ or O_3_

Number of Patients (NPT)	Day	PM_10_		O_3_	
	
		NPT change %	95% CI	NPT change %	95% CI
Total CVD; I00–I99	Concurrent	0.01	−0.05 to 0.07	−0.01	−0.23 to 0.20
33 458	Previous day	0.05	−0.01 to 0.11	0.23	0.02 to 0.44
	2 Cumulative*	0.03	−0.03 to 0.10	0.17	−0.06 to 0.40
	3 Cumulative^†^	0.04	−0.01 to 0.09	0.10	−0.15 to 0.35
-age < 15 year	Concurrent	−0.27	−0.76 to 0.19	−0.49	−2.05 to 1.07
681	Previous day	−0.15	−0.64 to 0.34	−1.06	−2.66 to 0.54
	2 Cumulative	−0.24	−0.76 to 0.28	−1.00	−2.80 to 0.80
	3 Cumulative	−0.09	−0.63 to 0.44	−0.82	−2.74 to 1.10
-age 15–64 year	Concurrent	−0.02	−0.12 to 0.07	−0.16	−0.47 to 0.15
16 710	Previous day	0.01	−0.08 to 0.10	0.01	−0.28 to 0.14
	2 Cumulative	−0.01	−0.10 to 0.09	−0.08	−0.43 to 0.26
	3 Cumulative	0.00	−0.09 to 0.10	−0.10	−0.47 to 0.27
-age ≥ 65 year	Concurrent	0.06	−0.03 to 0.16	0.15	−0.16 to 0.46
16 067	Previous day	0.10	0.03 to 0.19	0.50	0.19 to 0.81
	2 Cumulative	0.09	0.00 to 0.20	0.48	0.13 to 0.83
	3 Cumulative	0.08	−0.01 to 0.18	0.36	−0.01 to 0.73
Arrhythmia, I46–I49	Concurrent	−0.01	−0.30 to 0.29	−0.26	−1.24 to 0.72
1557	Previous day	−0.08	−0.37 to 0.21	−0.06	−1.00 to 0.88
	2 Cumulative	−0.05	−0.36 to 0.26	−0.20	−1.27 to 0.87
	3 Cumulative	−0.02	−0.34 to 0.29	−0.28	−1.43 to 0.87
Myocardial Infarction	Concurrent	0.12	−0.11 to 0.36	−0.30	−1.08 to 0.48
MI, I21	Previous day	0.00	−0.24 to 0.24	−0.68	−1.48 to 0.12
2566	2 Cumulative	0.06	−0.18 to 0.32	−0.68	−1.58 to 0.22
	3 Cumulative	−0.02	−0.27 to 0.22	−0.97	−1.95 to 0.01
Ischemic Heart Disease	Concurrent	0.02	−0.10 to 0.14	−0.30	−0.69 to 0.09
IHD, I20–I25	Previous day	0.07	−0.04 to 0.19	0.27	−0.12 to 0.66
10 158	2 Cumulative	0.05	−0.07 to 0.18	0.00	−0.45 to 0.45
	3 Cumulative	0.09	−0.01 to 0.20	−0.08	−0.57 to 0.41

*2cumulative = (lag0 + lag1)/2

^†^3cumulative = (lag0 + lag1 + lag2)/3

### The association between O_3_ and hospital visits for cardiovascular diseases

No association was found between ozone exposure and visits for total CVD (or for the arrhythmia, MI, or IHD) on the concurrent day (lag 0). However, after controlling for covariate factors, the total number of CVD visits increased by 0.23% (95% CI, 0.02–0.44) when the level of O_3_ was elevated on the previous day (lag 1). In addition, daily O_3_ concentration was positively associated with hospital visits for CVD among the elderly (≥65 years): a 0.50% (95% CI, 0.19–0.81) increase in hospital visits for CVD was associated with an increase in O_3_ during the previous day, and a 0.48% (95% CI, 0.13, 0.83) increase in hospital visits was associated with an elevation in the 2-day average O_3_ concentration (Table 3). We found no evidence of modifying effects on the results due to weather conditions.

## DISCUSSION

A large number of epidemiologic studies support an association between air pollution and hospitalization for CVD; however, most of these were conducted in cold or temperate climates with distinct seasonality.^1^^,^^2^^,^^6^^,^^22^^–^^24^ Few studies have been conducted in tropical climates with little seasonality. This study conducted in Bangkok is unique in its use of diagnostic codes to categorize patients with CVD who might be at heightened risk for hospital admission after exposure to pollutants. This study used time-series data on the health effects of PM_10_ and O_3_ concentrations associated with hospital visits in central Bangkok. The results demonstrated that exposure during the previous day to PM_10_ and O_3_ was positively associated with hospital visits for CVD among elderly patients.

A 10-µg/m^3^ increase in PM_10_ during the previous day was associated with a 0.10% (95% CI, 0.03–0.19) increase in daily hospital visits for CVD among elderly patients. The same increase in the 2-day average was associated with a 0.09% (95% CI, 0.00–0.20) increase in hospital visits among the elderly. In addition, a 10-µg/m^3^ increase in O_3_ during the previous day (lag 1) was associated with a 0.23% (95% CI, 0.02–0.44) increase in the total number of CVD visits and, among the elderly, a 0.50% (95% CI, 0.19–0.81) increase in daily hospital visits for CVD was observed after an increase in O_3_ during the previous day. The 2-day average level of O_3_ was more strongly associated with hospital visits among the elderly than was concurrent-day exposure (0.48% [95% CI, 0.13–0.83] vs 0.15% [95% CI, −0.16–0.46]) (Table 3).

Daily NO_x_ and CO concentrations in Bangkok during the period from 2002–2006 were obtained from PCD; these were far below levels believed to cause health effects and did not cause confounding.^42^ We also evaluated the effects of including NO_x_ or CO in multivariate analysis to examine the relation between hospital visits and PM_10_. The changes in the percentage increase in hospital visits for each 10-µg/m^3^ increase in PM_10_ were small, which suggests that the possibility of confounding by NO_x_ or CO was limited.^43^

The association between air pollution (PM_10_ and O_3_) and hospital visits for CVD in the elderly may be due to the fact that this group tends to be frail and may have pre-existing heart problems.^44^^,^^45^ The increase in hospital visits for CVD after a 10-µg/m^3^ increase in PM_10_ in elderly patients was lower than that found in cooler climates. Barnett et al^45^ studied 7 cities in Australia and New Zealand and reported a 1.1% (95% CI, 0.20–2.00) increase in hospital visits by the elderly after a 10-µg/m^3^ increase in PM_10_ and a 0.3% (95% CI, 0.10–1.00) increase in adult patients. A 2002 European Study (part of the Air Pollution and Health: A European Approach Project) examined the association between airborne particles and CVD hospital admissions (ICD 9th, 360–429), and found that the percentage increase in hospital admissions for a 10-µg/m^3^ increase in PM_10_ was 0.7% in the elderly (95% CI, 0.4–1.0).^24^ Unlike the majority of previous studies on air pollution and morbidity conducted in cities in the United States and Western Europe, which have relatively cold winters and strong seasonal patterns in daily morbidity, the present study was conducted in a tropical region and therefore excluded the effects of seasonal variation in climate. The replication in a tropical climate of findings obtained in cooler areas, albeit to a lesser extent, is noteworthy. In general, the level of air pollution and its effects on health are strongly associated with weather. In Japan, for example, mortality from CVD is 50% higher in the winter months than in the summer months.^46^ The results of the present study therefore show the net effect of air pollution, by excluding the effects of seasonality. However, human O_3_ exposure has been associated with a decrease in HRV,^13^ but there have been conflicting reports on O_3_ exposure and hospital admissions for CVD. Koken et al^17^ compared air pollution exposure with daily CVD hospital admissions among elderly people in Denver, Colorado in the United States. The results suggested that O_3_ is associated with an increased risk of hospitalization for acute myocardial infarction, coronary atherosclerosis, and pulmonary heart disease. The results show that there was no significant association between air pollution (PM_10_ and O_3_) and patients treated for the diagnostic subcodes for arrhythmia, MI, and IHD. This may be due to the limited number under subcodes for CVD. In fact IHD, which is the largest component of CVD, was weakly associated with PM_10_ exposure during the previous day.

Wong et al^22^ conducted parallel analyses of the short-term association between air pollution and daily hospital admissions in Hong Kong and London. The association between O_3_ and cardiac admissions was negative in London but positive in Hong Kong. In general, the effects of gaseous pollutants on CVD have not been systematically examined, and the mechanisms by which pollutants affect cardiovascular health continue to be a matter of speculation.

There were some limitations in the current study. First, although PM_10_ data were used in this time-series analysis, PM_2.5_ particulate matter with an aerodynamic diameter of less than 2.5 µm is more toxic and should therefore be analyzed in future studies. Second, we considered ambient environmental pollution rather than individual exposure levels in a cross-sectional study of a wide range of populations, which may have resulted in the omission of some extreme exposure incidents in our study. The use of ambient PM_10_ levels from PCD stations, rather than levels of personal exposure to PM_10_, may result in misclassification of exposure. This possible misclassification may not differentiate between those visiting hospitals and those not visiting, so that estimation of the association between air pollution and hospital visits may be biased toward null and thus underestimate the effects.^38^ Despite this possible bias, we nevertheless found effects due to exposure to PM_10_ and O_3_ and perhaps these would have been stronger had we been able to measure the actual individual exposure for each. Third, when looking at the acute health effects of air pollution using hospital visits, the availability and accessibility of health services often distort the real picture and this may have happened in the present study. However, in 2001, the Primary Care Unit System (with visits fixed at 30 Thai baht per hospital visit) was introduced in Thailand. This reduced the economic barrier to hospital treatment and allowed the number of hospital visits caused by air pollution to be assessed more directly. There may be still other limitations inherent to epidemiologic studies using government and hospital statistics; however, we believe that the present study showed a credible association between hospital visits for CVD and air pollution.

In conclusion, we found that PM_10_ and O_3_ were associated with cardiovascular diseases, particularly among elderly patients living in central Bangkok. There was a short-term association between increases in daily levels of air pollutants and the number of emergency hospital visits for CVD per day, particularly among individuals aged 65 years or older.
